# Implementation of New TB Screening Requirements for U.S.-Bound Immigrants and Refugees — 2007–2014

**Published:** 2014-03-21

**Authors:** Drew L. Posey, Mary P. Naughton, Erika A. Willacy, Michelle Russell, Christine K. Olson, Courtney M. Godwin, Pamela S. McSpadden, Zachary A. White, Terry W. Comans, Luis S. Ortega, Michael Guterbock, Michelle S. Weinberg, Martin S. Cetron

**Affiliations:** 1 Division of Global Migration and Quarantine, National Center for Emerging and Zoonotic Infectious Diseases, CDC

For more than two decades, as the number of tuberculosis (TB) cases overall in the United States has declined, the proportion of cases among foreign-born persons has increased. In 2013, the percentage of TB cases among those born outside the country was 64.6%. (1). To address this trend, CDC has developed strategies to identify and treat TB in U.S.-bound immigrants and refugees overseas. Each year, approximately 450,000 persons are admitted to the United States on an immigrant visa, and 50,000–70,000 are admitted as refugees. Applicants for either an immigrant visa or refugee status are required to undergo a medical examination overseas before being allowed to travel to the United States. CDC is the federal agency with regulatory oversight of the overseas medical examination, and panel physicians appointed by the U.S. Department of State perform the examinations in accordance with Technical Instructions (TI) provided by CDC’s Division of Global Migration and Quarantine (DGMQ). Beginning in 1991, the algorithm for TB TI relied on chest radiographs for applicants aged ≥15 years, followed by sputum smears for those with findings suggestive of TB; no additional diagnostics were used. In 2007, CDC issued enhanced standards for TB diagnosis and treatment, including the addition of sputum cultures (which are more sensitive than smears) as a diagnostic tool and treatment delivered as directly observed therapy (DOT). This report summarizes worldwide implementation of the new screening requirements since 2007. In 2012, the year for which the most recent data are available, 60% of the TB cases diagnosed were in persons with smear-negative, but culture-positive, test results. The results demonstrate that rigorous diagnostic and treatment programs can be implemented in areas with high TB incidence overseas.

## 2007 Technical Instructions

In 2007, CDC issued Technical Instructions for Tuberculosis Screening and Treatment Using Cultures and Directly Observed Therapy (CDOT TB TI).* Important changes included requiring 1) sputum cultures in addition to sputum smears; 2) tuberculin skin tests or interferon gamma release assays (beginning in 2009) for certain children aged 2–14 years examined in countries where the World Health Organization estimated TB incidence is ≥20 per 100,000 persons; 3) drug-susceptibility testing of positive isolates; and 4) treatment according to guidelines from the American Thoracic Society, CDC, and the Infectious Diseases Society of America (Figure). Treatment is delivered as DOT (a trained health-care professional administers and documents each dose) throughout the entire course.

## Implementation of CDOT TB TI

CDC’s DGMQ initially targeted large-volume, high TB-incidence countries to implement CDOT TB TI. Within each screening country, DGMQ worked to develop the infrastructure for implementing sputum culture and DOT. As TB diagnostic and treatment capacities were being developed, DGMQ linked panel physicians with in-country TB programs. A training program for panel physicians was developed that used multiple training modalities, including onsite instruction, webinars, development of a panel physician website,† and regional training summits. To monitor and evaluate the implementation and effectiveness of the algorithm, representatives of the Advisory Council for the Elimination of Tuberculosis and the National Tuberculosis Controllers Association led evaluations at five large-volume screening sites (Thailand, Philippines, Nepal, Vietnam, and Dominican Republic). In addition, to assist these programs with monitoring and evaluating their own progress, the TI require panel physicians to report statistical indicators of their medical screening process.

The first screening programs to implement CDOT TB TI were the refugee screening program in Thailand (for Hmong and Burmese refugees) on April 9, 2007, and the immigrant screening programs in Mexico and Philippines on October 1, 2007. Each year, panel physicians in additional countries conducted the screening according to the new standards. By 2011, panel physicians were using the new instructions in 51 countries, screening 70% of immigrant arrivals to the United States. By August 2012, DGMQ had worked with the Bureau of Consular Affairs at the U.S. Department of State to establish a deadline of October 1, 2013, for panel physicians worldwide to be screening in accordance with CDOT TB TI. That deadline was met by nearly all countries.

## Results of Implementation

During 2007–2013, site visits were conducted in 71 of the 151 jurisdictions that have panel physicians. To fulfill the laboratory culture requirement, new laboratories performing TB cultures were developed in China, India, Kenya, Mexico, Nepal, Thailand, Vietnam, and other countries. In addition, laboratories serving panel physicians in several countries developed the capability to perform drug-susceptibility testing on second-line drugs, which are used to treat multidrug resistant TB (MDR TB). These countries include China, Kenya, Nepal, Thailand, and Vietnam. During 2008–2013, 10 training summits were conducted, attended by panel physicians or U.S. Department of State consular officers, representing a total of 101 countries.

Preliminary analysis of crude indicators reported by panel physicians indicated that approximately 1,100 cases of TB were diagnosed during 2012, the year for which the most recent data were available. Approximately 60% of all cases were smear-negative, but culture-positive. Because the previous system did not require cultures, the smear-negative but culture-positive cases represent a gain in TB diagnoses with the new CDOT TB TI requirements. Of the cases diagnosed during 2012, 14 were MDR TB.

What is already known about this topic?

The United States is one of the largest immigrant and refugee–receiving countries. Preimmigration screening for tuberculosis (TB) historically has been required before entry and has been demonstrated as effective in preventing importation of active TB. However, the 1991 U.S. screening algorithm was outdated. New TB screening requirements, known as the Culture and Directly Observed Therapy Technical Instructions (CDOT TB TI), were issued in 2007. CDOT TB TI use newer technologies and TB cultures to increase the diagnostic yield, and also require treatment in accordance with U.S. guidelines. Since 2007, CDC has been working to implement CDOT TB TI worldwide.

What is added by this report?

Implementation of CDOT TB TI is effectively complete. During 2007–2014, panel physicians began using the new screening algorithm in 147 of 151 jurisdictions. The diagnostic yield increased twofold to threefold, with approximately 1,100 cases of TB diagnosed worldwide during 2012; approximately 60% of these cases were smear-negative, but culture-positive, representing a gain in diagnostic yield with the new algorithm. Preliminary evidence suggests the percentage of persons with abnormal chest radiographs overseas, but negative sputum smears, who are diagnosed with TB upon arriving in the United States has decreased from approximately 7% to 1%–2%. Implementation also is temporally associated with a decline in reported cases of TB among foreign-born persons in the United States 1 year after their arrival.

What are the implications for public health practice?

Successful implementation of CDOT TB TI demonstrates that rigorous diagnostic and treatment programs meeting international standards can be implemented in areas with high incidence of TB overseas. To further reduce the number of cases of TB among foreign-born persons, consideration might be given to extending screening to long-term visitors, developing strategies to address latent TB infection in the foreign-born, and strengthening U.S. follow-up for arriving persons identified overseas as being at risk for TB.

### Discussion

Overseas implementation of CDOT TB TI during April 2007–February 2014 was a successful worldwide TB intervention that directly benefitted U.S. TB control. In addition to increasing the yield of diagnoses overseas, implementation of CDOT TB TI was temporally associated with a decline in TB cases among foreign-born persons in the United States since 2007 (2). Although many factors could have produced the decline in TB rates, an increase in diagnosis and treatment of active TB overseas and the timing of the decline suggests implementation of CDOT TB TI was a major determinant (DGMQ, CDC, unpublished data, 2014).

Screening applicants for U.S. immigration status has been demonstrated to be an effective tool for identifying persons with TB disease before they enter the United States (3). However, given the nature of TB, vigilance after arrival also is needed, because persons with latent TB infection can convert to an active state after arrival. During the period in which the 1991 TB TI was in use, 7% of immigrants and refugees who had abnormal chest radiographs suggestive of TB, but negative sputum smears, were diagnosed with TB disease after their arrival in the United States (3). Under CDOT TB TI, early data suggest that percentage has declined to 1%–2% (4). Although formal economic analyses have not been completed, the gains in overseas diagnosis and the decrease in cases suggest that successful implementation of this screening program could result in crude savings in excess of $15 million yearly.

A previous analysis determined that investments in TB control in countries where the disease is endemic can yield a greater return on investment than only improving preentry screening algorithms (5). For this reason, a key component of DGMQ’s implementation plan has been to link panel physicians with their country’s national TB programs. Such successful linkages have included panel physicians in the Dominican Republic, who entered into a public-private partnership with that country’s National Tuberculosis Program (6), and the International Organization for Migration, which manages refugee resettlements and serves as the panel physician for applicants in Nairobi, Kenya, providing laboratory testing and DOT for certain nonresettling or immigrating populations (International Organization for Migration, unpublished data, 2011). To maximize the opportunity for the laboratory and treatment infrastructure to benefit more than U.S.-bound populations, efforts should continue to seek ways in which the screening program can collaborate with in-country TB programs.

Although the 1991 algorithm was shown to help prevent importation of TB (2), it did not incorporate newer, more sensitive technologies for diagnosing TB or include a treatment component. To help determine what changes could be made to the TI, DGMQ and CDC’s Division of Tuberculosis Elimination collaborated on research activities. A key outcome of that effort was the 2006 publication of a study demonstrating that, compared with the gold standard of mycobacterial cultures, the 1991 algorithm relying on sputum smears was only 34% sensitive in diagnosing TB (7).

Implementation of CDOT TB TI is one part of a broader strategy to address TB among foreign-born persons in the United States. Resources should be devoted toward rigorous monitoring of the program to maintain what has been developed and increase linkages with in-country efforts. Moreover, additional strategies to further decrease TB among foreign-born persons might be explored, such as extending screening to long-term visitors (8), developing innovative strategies to address the reservoir of latent TB infection in the foreign-born population (9), and strengthening U.S. follow-up for arriving persons identified overseas as being at risk for TB (10).

## Figures and Tables

**FIGURE:**
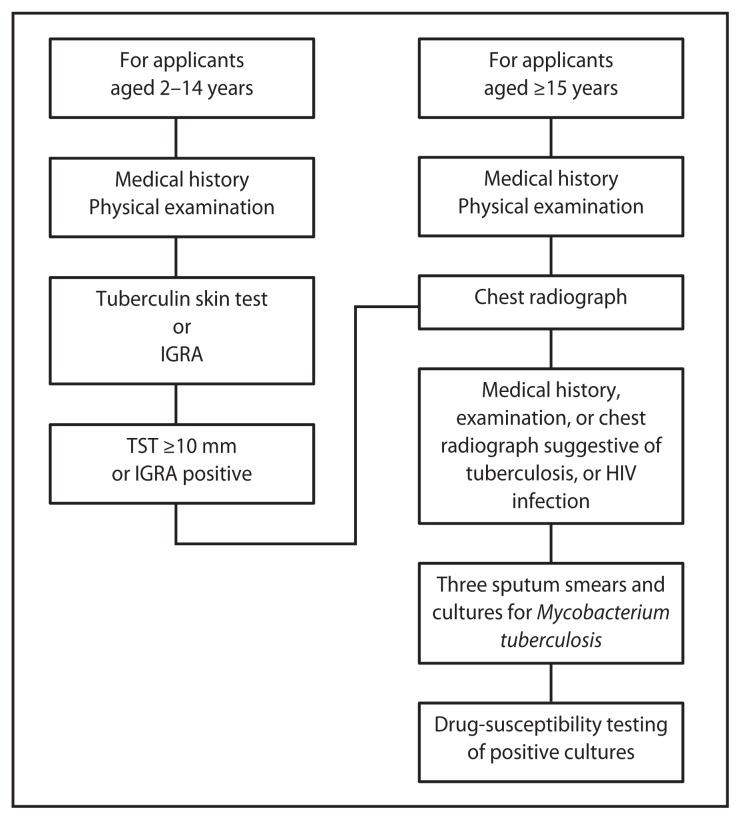
Tuberculosis screening algorithm for applicants aged ≥2 years in countries with a tuberculosis incidence rate estimated by the World Health Orgnization at ≥20 cases per 100,000 population — Technical Instructions for Tuberculosis Screening and Treatment Using Cultures and Directly Observed Therapy,^*^ United States, 2009 **Abbreviations:** IGRA = interferon gamma release assay; TST = tuberculin skin test; HIV = human immunodeficiency virus. ^*^ Available at http://www.cdc.gov/immigrantrefugeehealth/pdf/tuberculosis-ti-2009.pdf.

## Footnotes

* Available at http://www.cdc.gov/immigrantrefugeehealth/pdf/tuberculosis-ti-2009.pdf.

† Available at http://www.cdc.gov/panelphysicians/index.html.
